# Supplementary material for: Temporal integration of auxin information for the regulation of patterning
Source: eLife. 2020 May 7;9:e55832. doi: 10.7554/eLife.55832 (PMC7205470; doi:10.7554/eLife.55832)
Supplement: Supplementary file 1. [file elife-55832-supp1.docx]

**Supplementary File 1. RNAseq expression of YUCCA genes at the SAM**

| **AGI** | **Name** | **TPM Col Rep1** | **TPM Col Rep2** | **TPM Col Rep3** | **TPM Col mean** |
| --- | --- | --- | --- | --- | --- |
| **AT5G11320** | **YUC4** | 7.56 | 10.31 | 12.17 | 10.02 |
| **AT4G32540** | **YUC1** | 8.56 | 8.59 | 11.98 | 9.71 |
| **AT5G25620** | **YUC6** | 1.50 | 2.37 | 1.94 | 1.94 |
| **AT4G13260** | **YUC2** | 0.68 | 0.77 | 0.55 | 0.67 |
| **AT1G04610** | **YUC3** | 0.05 | 0.08 | 0.15 | 0.09 |
| **AT4G28720** | **YUC8** | 0.06 | 0.06 | 0.09 | 0.07 |
| **AT1G04180** | **YUC9** | 0 | 0 | 0 | 0 |
| **AT2G33230** | **YUC7** | 0 | 0 | 0 | 0 |
| **AT5G43890** | **YUC5** | 0 | 0 | 0 | 0 |
| **AT1G48910** | **YUC10** | 0 | 0 | 0 | 0 |
| **AT1G21430** | **YUC11** | 0 | 0 | 0 | 0 |

Transcripts Per Kilobase Million (TPM). Data from *(64)*
